# Identifying key determinants of health among China’s migrant population using machine learning methods: Evidence from the china migrants dynamic survey

**DOI:** 10.1371/journal.pone.0335168

**Published:** 2025-11-10

**Authors:** Bo Dong, Yuxin Zhou, Li Wang, Yiyu Wang, Zhenlin Zhang

**Affiliations:** 1 School of Public Health, Zhejiang Chinese Medicine University, Hangzhou, Zhejiang Province, China; 2 School of Marxism, Xi’an Jiaotong University, Xi’an, Shaanxi Province, China; 3 School of nursing, Qilu Medical University, Zibo, Shandong Province, China; 4 J.E. Cairnes School of Business & Economics, University of Galway, University Road, Galway, Ireland; 5 China Jiliang University College of Modern Science and Technolog, Yiwu, Zhejiang Province, China; University of Washington, UNITED STATES OF AMERICA

## Abstract

**Background:**

Continuously improving health security for the migrant population is a key component of China’s healthcare system reform. Existing research indicates that migrant health is influenced by multiple factors, yet the relative importance of these factors remains inadequately measured. This study aims to analyze the current health status of China’s migrant population and rank the primary factors influencing their health based on importance.

**Methods:**

Data were sourced from the 2018 China Migrants Dynamic Survey, including 108,669 cases after data cleaning. The health status of the migrant population was initially analyzed using frequency and percentage distributions. Logistic regression was then applied to examine the relationship between various factors and migrant health. Subsequently, six machine learning methods (Neural Network, Random Forest, Support Vector Machine, Gradient Boosting Machine, Extra Trees, and Decision Tree) were applied to rank the importance of these factors. A multidimensional performance metric system (accuracy, precision, recall, F1 score, and AUC value) was employed to comprehensively evaluate the classification performance of the models. SHAP (Shapley Additive Prediction) values were used to illustrate the contribution of different factors to the health status of the migrant population.

**Results:**

The health status of China’s migrant population is generally positive, though it is influenced by multiple factors, with varying degrees of significance. Among six distinct machine learning models, the Random Forest model demonstrated the best predictive performance. Its results indicate that the key factors affecting migrant health are age, employment, income, and education level. SHAP value analysis reveals that stable employment, higher education levels, and higher income are positively correlated with better health outcomes, while age was predominantly negatively correlated, indicating a detrimental effect on health status.

**Conclusion:**

The overall health status of China’s migrant population is relatively optimistic. However, their disadvantaged positions in areas such as education and income expose them to higher health risks. To address these key determinants, further improvements in health safeguards should focus on: developing stratified intervention strategies based on age structure differences; optimizing work environments and employment security; enhancing health literacy; and strengthening public health emergency management and social support systems.

## Introduction

In China, internal migrants are defined as individuals whose current place of residence does not align with their household registration, commonly known as Hukou [1]. The migrant population has made indelible contributions to China’s rapid economic development [2,3], and migration has become an important survival strategy for millions of rural residents [4]. China also has the largest migrant population in the world. According to the Seventh National Population Census released by the Chinese government in 2021, the number of migrants in China reached nearly 380 million in 2020—an increase of 150 million compared to 2010, representing a growth rate of almost 70% over the decade [5]. While migrant populations have injected strong momentum into local development and gained significant opportunities for advancement [6]. However, as the scale and frequency of migration increase, concerns over health security for these populations are growing.

In July 2022, the World Health Organization published the World Report on Refugee and Migrant Health, which emphasized that millions of refugees and migrants are in highly vulnerable situations [7], often experiencing poorer health outcomes than host country residents. Furthermore, due to changes in their registered place of residence, migrant groups may lose social capital and face discrimination in employment opportunities and social welfare rights [8]. Simultaneously, they are often excluded from local healthcare systems, unable to fully utilize existing health service resources [9]. This results in migrant populations facing unequal treatment compared to residents in accessing public social services, making it difficult to meet their health needs. This creates additional health risks and leads to numerous health inequities. Moreover, the global migrant population continues to expand. Research indicates approximately 2.3% of the world’s population resides outside their country of nationality. This equates to roughly 281 million international migrants and 89 million forced migrants, with these figures steadily rising over recent decades [10,11]. The proportion of immigrants in high-income countries is also steadily rising, particularly in the United States and Europe. To address these concerns and improve the health of migrant populations, numerous developed countries and regions have established public health service regulations and implemented management programs addressing immigrants’ employment and living conditions [12–14]. For instance, France provides medical assistance to undocumented immigrants through the AME (National Health Assistance) program [15]; the United States protects migrant health through initiatives such as immigrant health screening and monitoring, expanding Medicare and Medicaid programs, and the implementation health promotion and education initiatives targeting migrant populations [16].

Similar to the lower socioeconomic status and higher health risks faced by migrant populations abroad, research in China also indicate that the majority of migrant population migrates from rural to urban areas. They possess low educational attainment, engage in labor-intensive work, and endure poor working environments and living conditions [3,17]. Consequently, they are particularly vulnerable to occupational diseases, infectious diseases, sexual and reproductive health issues, maternal health complications, and psychological disorders [18,19]. To address these challenges, China has implemented multiple public health interventions. In 2013, it launched the Equalization of Basic Public Health and Family Planning Services for Migrant Populations Project. This aims to enhance equitable access to public health services for migrants by conducting health education, establishing comprehensive health records, and improving healthcare accessibility, thereby narrowing health disparities and inequalities. In 2016, China introduced the “Healthy China 2030 Plan Outline” to advance the Healthy China initiative and elevate national health standards. This policy explicitly states that building a Healthy China requires “promoting equitable access to basic public health services, safeguarding the public welfare nature of fundamental medical and health services, gradually narrowing disparities in basic health services and health levels across urban-rural areas, regions, and populations, and achieving universal health coverage.” Currently, policies for equalizing basic public health services continue to advance incrementally. These policies have demonstrated significant positive impacts on the health of migrant populations, enhancing their overall health status and narrowing the health gap between migrants and residents [20,21].

The health of migrant populations is influenced by multiple factors, including age, income, educational attainment, and public health policies. For instance, Min et al. indicated that household income has a significant positive impact on the health of elderly migrants [22]. Fan argued that the generally low educational level among migrants leads to insufficient health awareness and understanding of disease consequences, thereby affecting their health status [23]. Beyond these, numerous studies also indicate that gender, marital status, household registration status, employment conditions, and duration of migration significantly affect the health status of migrant populations [24,25]. Ruhnke et al. found that undocumented immigrants in the United States, as a particularly vulnerable group, face numerous additional economic and cultural risk factors affecting their health [26]. Afrin et al. revealed that the survival of the migrant population in Dhaka, Bangladesh, depends on a complex fusion of coping and adaptation measures rooted in their social relations, communal living, and mental resilience [27]. Holguin et al. noted that host countries’ capacity to address migrants’ healthcare needs (e.g., mental health, childhood immunizations, chronic disease management) is significantly diminished. This situation is often exacerbated by factors including: difficulties in identifying the inherent challenges of migrant populations, insufficient awareness among health officials regarding migrant needs, lack of targeted and comprehensive response strategies, and limited available resources [9]. Additionally, public services such as health insurance and health education influence migrant health. Meng et al. found that health insurance enrollment significantly improves health outcomes among elderly migrants [28]. He et al. demonstrated that health education helps middle-aged and elderly migrants establish health records and enhance their health levels [29].

Beyond the above factors, public health crises significant impact the health of migrant populations. During the COVID-19 pandemic, in particular, migrant populations—as marginalized groups—faced substantial health challenges. Studies from China and other countries consistently demonstrate a close and complex interplay between public health crises and the health status of migrant populations [30–34]. Migrant populations often face heightened vulnerability during public health crises due to socioeconomic status, living and working conditions, cultural and language barriers, and limited access to healthcare resources [35,36]. A telephone survey conducted in China in July 2020 on public perceptions of social policy responses during the pandemic revealed that migrant workers experienced significantly lower rates of assistance when unemployed during the outbreak, enduring more severe hardships. Fragmented social security systems and limited portability of welfare policies left many vulnerable migrants without essential social protection [37]. Vaccine acceptance is critical for addressing public health threats posed by COVID-19 and other preventable vaccine-preventable diseases. Relevant studies also indicate relatively low vaccination rates among migrant populations, underscoring the need to further enhance vaccine confidence within this group. A cross-sectional study in Shanghai, China, in November 2020 assessed COVID-19 vaccine acceptance and their willingness to pay among migrant workers. While acceptance was relatively high, concerns about safety, efficacy, and cost emerged as potential barriers to vaccination [38]. Daniels et al. conducted a meta-analysis of 22 studies examining vaccine hesitancy among the U.S. migrant population. They concluded that vaccination rates among migrants are significantly lower than those in the general U.S. population. Key barriers include limited health knowledge, difficulties in accessing services, and low trust in the healthcare system [39]. Loganathan et al. analyzed the adverse impacts of the COVID-19 pandemic on Malaysian migrant populations through in-depth interviews. Findings revealed that economic hardship impeded migrants’ ability to purchase high-quality masks, while they also lacked awareness of the importance of non-pharmaceutical interventions in disease prevention. Many migrants avoided government-designated isolation centers due to financial constraints and fear of arrest, with delayed medical treatment potentially contributing to higher COVID-19 mortality rates among this population [40].

Regarding methodologies for studying health determinants among migrant populations, existing research primarily employs methods like logistic regression to examine whether and to what extent these factors influence health outcomes [41,42]. While this approach identifies correlations between various factors and migrant health, it fails to assess the relative importance of different factors within these relationships. Ensuring migrant health requires identifying and prioritizing the most significant promoting or hindering factors. Most previous studies rely on traditional methods, potentially failing to capture the cumulative effects of multiple factors and thus limiting the accuracy of their relative importance assessments. In contrast, machine learning offers a promising alternative approach for more effectively identifying and prioritizing key determinants by analyzing large, complex datasets. Machine learning algorithms can detect intricate patterns and relationships among variables that traditional methods may overlook, demonstrating potential for risk factor ranking across multiple health domains.

In summary, migrant populations represent a distinct group with significant public health implications worldwide. While scholarly research has extensively addressed the health status of these populations, several gaps remain. First, the subject matter remain relatively limited; most scholars confine their investigations to specific subgroups, such as middle-aged and elderly migrants, without comprehensively examining the health determinants affecting the entire migrant population. Second, methodological approaches are often limited to analyzing isolated factors without comparing the relative importance of multiple variables. Given this, and considering the unique circumstances faced by China’s migrant population—characterized by large-scale mobility, high frequency of movement, and inadequate health policy safeguards—this study investigates the determinants of migrant health using representative large-scale cross-sectional data from the China Migrants Dynamic Survey. Six machine learning methods (Neural Network, Random Forest, Support Vector Machine, Gradient Boosting Machine, Extra Trees, and Decision Tree) are applies to rank the most significant characteristics affecting migrant health, aiming to provide valuable insights for developing targeted prevention and intervention strategies.

Compared to existing research, the marginal contributions of this study are as follows. First, the sample is expanded to cover the entire migrant population. It is estimated that over 96% of migrants are under 60 years old [43], an age group that typically exhibits health behaviors distinct from those of the elderly [44]. Therefore, extending the research sample to the entire migrant population lends greater generalizability to the findings and provides more robust theoretical foundations for improving health security policies for migrants. Second, this study employs multiple machine learning models to measure the importance of factors influencing migrant health and identifies the most critical ones. This facilitates more accurate assessments of these factors, enabling the development of targeted measures to improve health outcomes. Third, the large-scale, multidimensional analysis based on CMDS data provides robust evidence-based support for the Chinese government in formulating health policies for the migrant population. International research indicates that comprehensive empirical studies underpinning policy development have become a significant trend in public health and social policy. The innovative application of this study within China’s unique national context enhances the effectiveness and precision of public health policy interventions, advancing the refinement of health service systems for the migrant population during urbanization. Furthermore, the methodologies and research framework employed in this study demonstrate strong replicability and scalability. They can be applied not only to health research on other populations or regions within China but can also be extended to immigrant health studies in other countries and regions, thereby carrying broader international academic influence and practical significance. It is anticipated that this study will not only contribute to refining health protection policies for migrants but also serve as a reference for enhancing the health status and optimizing policies for migrant populations in other nations.

## Research methodology

### Theoretical framework

**Fig 1:**
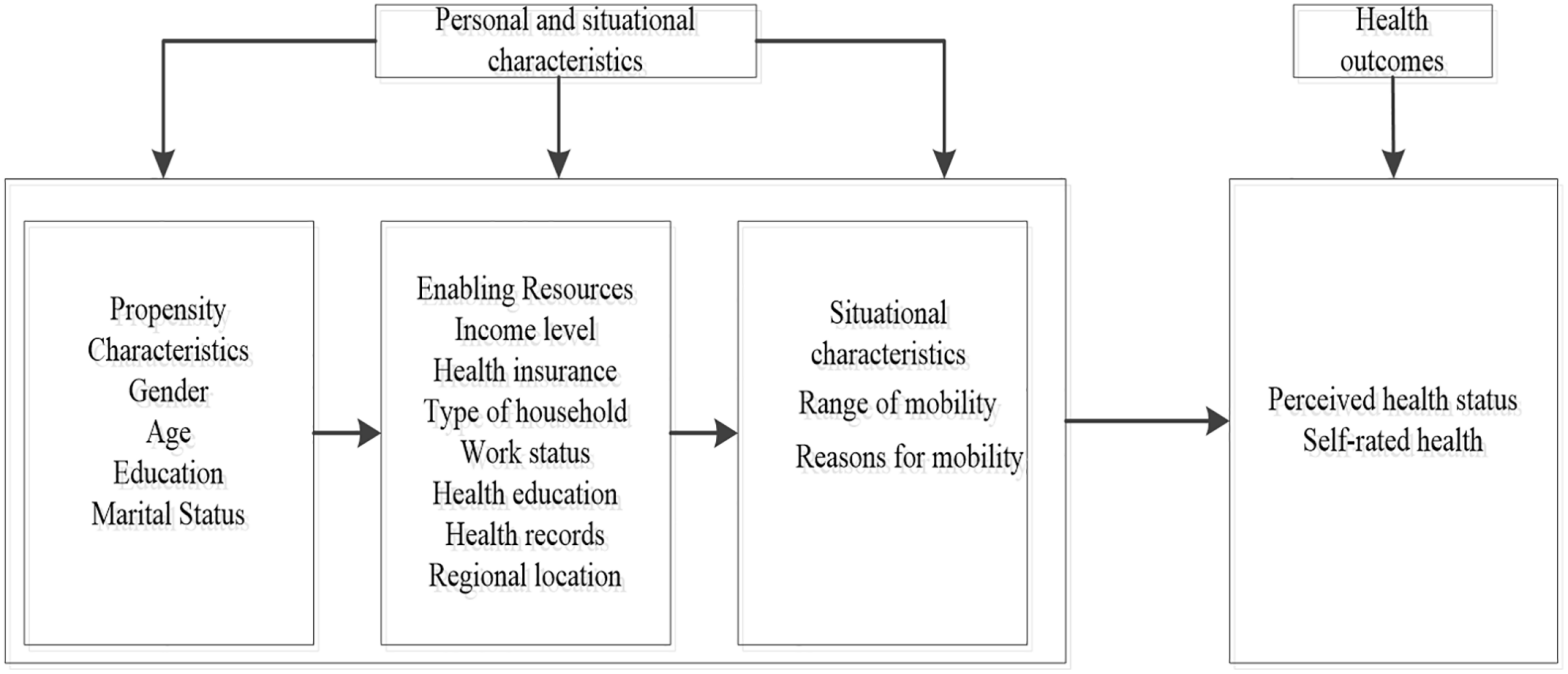
Framework for analyzing health determinants of migrant populations based on the anderson model.

The theoretical framework further clarifies the determinants of migrant health. Currently, Anderson’s model is widely recognized as a key theoretical framework for examining health service utilization behavior, and it has been extensively applied in healthcare service research [45,46]. It has also been utilized in studies focusing on public health, healthcare service utilization, and migrant health [47,48]. This study adopts this model as its theoretical framework. Based on the characteristics of the migrant population and relevant literature [49,50], a theoretical framework for analyzing the determinants of migrant health was constructed across three dimensions: predisposing factors, enabling factors, and contextual factors, as illustrated in Fig 1.

### Data sources

The data were obtained from the 2018 China Migrants Dynamic Survey (CMDS). This project represents a large-scale, nationwide cross-sectional questionnaire survey conducted annually by China’s National Health Commission since 2009, targeting the migrant population [1]. CMDS employs a multistage stratified probability proportional to size (PPS) cluster sampling strategy. In the first stage, townships (towns, subdistricts) are selected using PPS sampling. In the second stage, PPS sampling is applied to select villages (neighborhood committees) within the sampled townships (towns, subdistricts). The third stage involves selecting individual respondents from the sampled villages (neighborhood committees). The survey also employs rigorous methods to ensure data quality, including scientifically designed questionnaires, training for interviewers, deployment of supervisors to verify questionnaires, and quality checks through telephone follow-ups.

The 2018 CMDS survey sample was drawn from the 2017 China Migrant Population Information System, including 152,000 respondents across 32 provincial-level administrative regions. The target population comprised migrants aged 15 or older residing outside their registered domicile (urban or rural counties) for over one month. Therefore, this study defines the migrant population as those residing in their place of residence for one month or longer without local residence registration. The CMDS survey content not only includes the demographic and socioeconomic characteristics of respondents and their family members but also covers their health status and utilization of public health services. For more details on the sample, please refer to other literature [33]. After handling missing data values, the final sample size included in this study’s analysis was 108,669.

### Variables selection

#### Dependent variables.

In this study, self-rated health was used to measure the health status of the migrant population. Prior study has confirmed that self-assessed health is generally consistent with an individual’s actual health condition [51, 52], making it a widely applicable indicator and a reliable proxy for objective health status [53]. Respondents were asked the question: “How is your health?” with four possible answers: unable to care for oneself, unhealthy but able to care for oneself, basically healthy, and healthy. For analytical purposes, these responses were consolidated into three categories: “1 = Unhealthy” (including unable to care for oneself and unhealthy but able to care for oneself), “2 = Basically Healthy,” and “3 = Healthy.” Higher values indicate better health status.

#### Independent variables.

Based on the analytical framework for health determinants among migrant populations, this study selected independent variables encompassing propensity characteristics, enabling resources, and demand characteristics. Propensity characteristics include gender, age, education, and marital status. Enabling resources include household registration status, medical insurance coverage, income, employment status, access to health education, health records, and geographic region. Demand characteristics primarily involve migration reasons and migration scope. Given the selected data conditions, specific settings are as follows: Gender is a categorical variable, with males coded as 1 and females as 2; Age is calculated as the difference between the month/year of interview and the month/year of birth; Educational attainment is a 0–5 level variable based on individual academic qualifications: never attended school = 1, elementary school = 2, junior high school = 3, senior high school = 4, college or above = 5; marital status is a categorical variable where individuals in first marriage, remarriage, or cohabitation are coded as 1, while unmarried, divorced, or widowed individuals are coded as 2; employment status is defined as employed = 1, unemployed = 2; household registration is a categorical variable where urban = 1, rural = 2; medical insurance is categorized as enrolled = 1, not enrolled = 2; Income level: Household income data converted into provincial rankings (<20th percentile, 20%−39th percentile, 40%−59th percentile, 60%−79th percentile, ≥ 80th percentile) for analysis. Health education: Received health education = 1, did not receive = 2. Health records: Binary variable—established health records = 1, no records established = 2. The region is categorized as Western, Central, and Eastern, assigned values 1, 2, and 3, respectively. Migration scope is divided into three categories: inter-city migration = 1, inter-provincial migration = 2, inter-county migration = 3. Migration reasons are also classified into three categories: work-related migration = 1, other reasons = 2, family reasons = 3. Variable definitions and assignments are detailed in Table 1.

**Table 1:** Variable definitions and assignments.

Variables		Definition
Dependent variable	Health	Unhealthy = 1
		Basic health = 2
		Health = 3
Independent variables	Medical insurance	Not participating = 1
		Participation = 2
	Gender	Female = 1
		Male = 2
	Age	15-30 = 1
		31-45 = 2
		46-60 = 3
		61+=4
	Educational level	Illiteracy = 1
		Primary school = 2
		Junior high = 3
		High school = 4
		University = 5
	Marriage	Unmarried = 1
		Married = 2
	Work	Unemployment = 1
		Employment = 2
	Household registration	Rural = 1
		Urban = 2
	Range of mobility	Cross county = 1
		Cross-city = 2
		Cross-province = 3
	Reasons for mobility	Work = 1
		Family = 2
		Other = 3
	Income	Lowest (<percentile 20)=1
		Lower (percentile 20–39)=2
		Middle (percentile 40–59)=3
		Higher (percentile 60–79)=4
		Highest (≥percentile 80)=5
	Health education	Not accepted = 1
		Accepted = 2
	Health record	Not established = 1
		Established = 2
	Region	West = 1
		Central = 2
		East = 3

### Analytical methods

#### Ordered logistic regression.

This study employs logistic regression analysis to examine the associations between multiple factors and migrant health. This methodology enables systematic identification of the impact of different factors on migrant health. Given that migrant health constitutes a three-category ordinal variable, an ordered logistic regression model is utilized. The basic model is as follows:


$$P(Y=\text{1}|X\;)=\frac{\text{1}}{\text{1}+\text{exp}\left[-\left(\beta_{\text{0}}+\sum_{i=\text{1}}^{n}\beta_{i}x_{i}\right)\right]}$$

Where $P_{i}$ represents the probability that the migrant population is in a certain state of health, *Y* is the dependent variable (1 = Unhealthy, 2 = Basically Healthy, 3 = Healthy), *X* represents the independent variable affecting health, denoted as *n*, $X=(x_{\text{1}},x_{\text{2},}...,x_{n})$ is the regression intercept, $\beta_{\text{0}}$ represents the regression coefficient of the independent variable $\beta_{i}$, EXP(β) is the rate of change of the ratio of the probability of being healthy to that of being unhealthy,odds, which can be used as an indicator of the estimated size of the effect to measure the magnitude of the impact of a certain independent variable. odds is the event occurrence ratio, which is calculated as follows:


$$odds=\frac{P(Y=\;\text{1}|X)}{P(Y=\;\text{0}|X)}=\frac{P}{\text{1}-P}$$

### Machine learning models

#### Model building.

To identify key determinants of migrant health, machine learning methods are employed to rank these factors by importance. Given the complexity and multifactorial nature of influencing factors, multiple machine learning algorithms are selected for comprehensive analysis, specifically including Neural Networks, Random Forests, Support Vector Machine, Gradient Boosting Machine, X-Tree, and Decision Trees. These algorithms each possess distinct advantages in pattern recognition and predictive modeling, making them particularly well-suited for analyzing the complex factors affecting migrant health. Additionally, these six machine learning methods are widely applied in studies evaluating the relative importance of influencing factors, with prior research confirming their robustness [54–67].

The training process involved several critical steps to ensure model validity and reliability. First, the dataset was randomly divided into a training set (70%) and a testing set (30%) using stratified sampling, facilitating model development and evaluation. This follows standard practice of explicitly separating data used for model learning from that used for evaluation, while maintaining proportional consistency across categories. The training set was used to fit each model, enabling the algorithms to ‘learn’ from the data patterns and relationships. Prior to model fitting, all features were standardized using StandardScaler, ensuring variables with different scales could be compared on a uniform scale. To ensure robust model performance, a 5-fold stratified cross-validation (StratifiedKFold) strategy we employed. During evaluation, the data was divided into five subsets, each serving as the validation set in turn, maintaining balanced category distribution for each validation pass. Hyperparameters for each algorithm were initialized with reasonable default values to balance accuracy and computational efficiency.

#### Model evaluation.

The performance of machine learning models is evaluated through key metrics to identify factors influencing migrant health and determine the most influential variables. This study compared six models, each providing valuable insights based on distinct data characteristics. These metrics are crucial for assessing model accuracy and reliability. The comprehensive and detailed metric system employed for evaluating machine learning models in this study is presented in Table 2, where Recall (Sensitivity) measures the proportion of true positive cases correctly identified.A high recall (≥0.80) in our study highlights the models’ effectiveness in predicting healthcare demand. Precision reflects the proportion of true positive predictions among all positive predictions, indicating the model’s ability to minimize false positives. The F1 Score represents the harmonic mean of precision and recall, providing a balanced evaluation that accounts for both false positives and false negatives. High F1 scores suggest reliable and balanced models. Accuracy was used to gauge the overall correctness of the models. The Area Under the ROC Curve (AUC) assesses a model’s ability to distinguish between classes, with higher scores indicating superior performance.

**Table 2:** Key metrics for machine learning.

Metric	Definition	Formula
Recall (Sensitivity)	The proportion of actual positives correctly identified by the model. Indicates how well the model captures positive instances.	Recall = TP/ (TP + FN)
Precision	The proportion of positive predictions that are actually correct. Reflects the accuracy of positive predictions.	Precision = TP/ (TP + FP)
F1 Score	The harmonic mean of precision and recall. Balances the trade-off between precision and recall.	F1 Score = 2 * (Precision * Recall)/ (Precision + Recall)
Accuracy	The proportion of correctly predicted samples to the total number of samples. This metric reflects the overall accuracy of the model’s predictions and provides an intuitive measure of its performance.	Accuracy = (TP + TN)/ (TP + TN + FP + FN)
AUC	Measures the area under the Receiver Operating Characteristic curve. Evaluates the model’s ability to distinguish between classes.	–

Note: TP (True Positive): The number of samples correctly predicted as positive by the model; TN (True Negative): The number of samples correctly predicted as negative by the model; FP (False Positive): The number of samples incorrectly predicted as positive by the model; FN (False Negative): The number of samples incorrectly predicted as negative by the model.

To further validate the statistical significance of the models, we implemented the Bootstrap resampling method (100 resamples) to calculate the 95% confidence interval for accuracy. The DeLong test was used to statistically compare the AUC differences among different models. Additionally, the Hosmer-Lemeshow goodness-of-fit test was used to assess model calibration, ensuring consistency between predicted probabilities and actual outcomes. By integrating multiple algorithms, rigorous cross-validation strategies, and comprehensive statistical evaluation methods, a reliable and statistically significant predictive model is developed. This model effectively identifies and quantifies key features influencing the target variable, providing a robust foundation for subsequent feature importance analysis and clinical decision-making.

**Fig 2:**
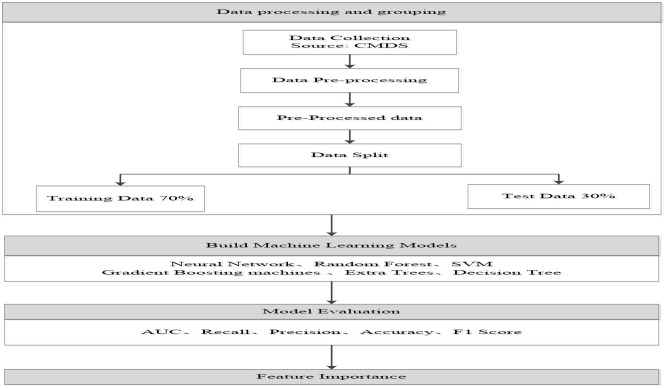
Study model for assessing the importance of different influencing factors.

Based on the above analysis, this study further employs a workflow diagram (Fig 2) to analyze the process of constructing and evaluating machine learning models using CMDS data. The workflow begins with data collection, followed by data preprocessing, including data cleaning and feature engineering. The processed data is then split into a training set (70%) and a test set (30%). Multiple machine learning models are trained using the training data and evaluated using metrics such as Recall, Precision, F1 Score, and Area Under the Curve (AUC) to determine their effectiveness. Additionally, feature importance analysis was conducted to identify the most influential features. This systematic process ensures thorough model evaluation and selection of the best-performing model while providing deep insights into the key factors driving model predictions.

### Statistical analysis

**Fig 3:**
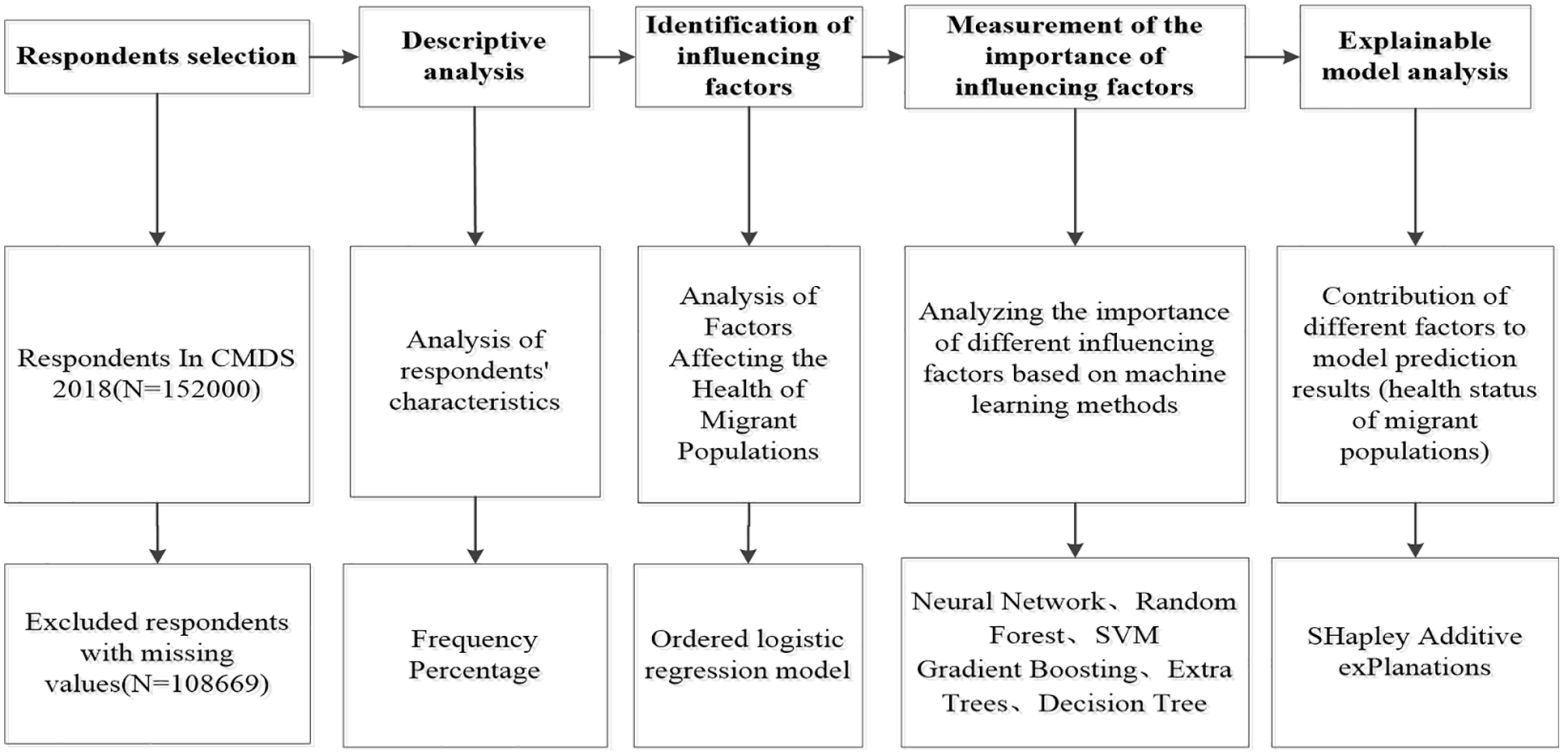
The flow chart of the study.

All statistical analyses were performed using Stata 22.0 and RStudio. First, categorical data were presented using case counts and percentages (%) to describe respondents’ basic characteristics. Subsequently, RStudio software was utilized for logistic regression and machine learning model analysis, yielding importance scores and rankings for different variable features. Finally, SHAP was applied to identify key features contributing to migrant health outcomes. The study workflow is illustrated in Fig 3.

### Ethics statement

As this study was a secondary analysis of de-identifified data collected by the government, ethics approval has been exempted. The 2018 China Migrants Dynamic Survey was approved by the China National Bureau of Statistics (No.Guotongzhi [2018] No.45), and written informed consent was obtained from all participants at the time of data collection. The use of the data for this study was approved by the Migrant Population Service Centre, National Health Commission of China. All procedures performed in this study were in accordance with the 1964 Helsinki declaration and its later amendments or comparable ethical standards. The datasets used in this study are publicly available and can be accessed via Migrant Population Service Center, National Health Commission of China. Requests to access these datasets should be directed to https://www.ncmi.cn/project/project-showProjectList.html?type=data&id=d3.

## Results

### Analysis of respondent characteristics

Table 3 reports the basic characteristics of the migrant population. The results indicate that 97.7% of migrants are in good health. More than 70% are enrolled in medical insurance. In terms of age distribution, 96.2% are under 60 years old, while only 3.8% are over 60; the educational attainment of migrants is relatively low, with 80.3% having completed high school or below. Regarding migration patterns, 47.9% migrated across provinces, and 83.8% migrated for work-related reasons. Other characteristics include: 63% of the migrant population had low-to-moderate income levels; 82.1% had received health education, but only 33.2% had established health records. At the regional level, 42.07% resided in the eastern region, while 29.54% and 28.39% lived in the central and western regions, respectively.

**Table 3:** Basic Characteristics of Respondents.

Variables		N	%
Health	Unhealthy	2507	2.3
	Basic health	12578	11.57
	Health	93584	86.12
Medical insurance	Not participating	32529	29.9
	Participation	76140	70.1
Gender	Female	53197	49
	Male	55472	51
Age	15-30	35875	33
	31-45	48493	44.6
	46-60	20140	18.5
	61+	4161	3.8
Educational level	Illiteracy	2660	2.4
	Primary school	14892	13.7
	Junior high	45760	42.1
	High school	23942	22.0
	University	21415	19.7
Marriage	Unmarried	17422	16
	Married	91247	84
Work	Unemployment	18561	17.1
	Employment	90108	82.9
Household registration	Rural	73623	67.7
	Urban	35046	32.3
Range of mobility	Cross county	19617	18.05
	Cross-city	36999	34.05
	Cross-province	52063	47.9
Reasons for mobility	Work	91080	83.8
	Family	15617	14.4
	Other	1972	1.8
Income	Lowest (<percentile 20)	24032	22.1
	Lower (percentile 20–39)	22260	20.5
	Middle (percentile 40–59)	22149	20.4
	Higher (percentile 60–79)	20916	19.2
	Highest (≥percentile 80)	19312	17.8
Health education	Not accepted	19416	17.9
	Accepted	89253	82.1
Health record	Not established	72600	66.8
	Established	36069	33.2
Region	West	30851	28.39
	Central	32105	29.54
	East	45713	42.07

### Analysis of the impact of different factors on migrant health

#### Correlation analysis.

**Fig 4:**
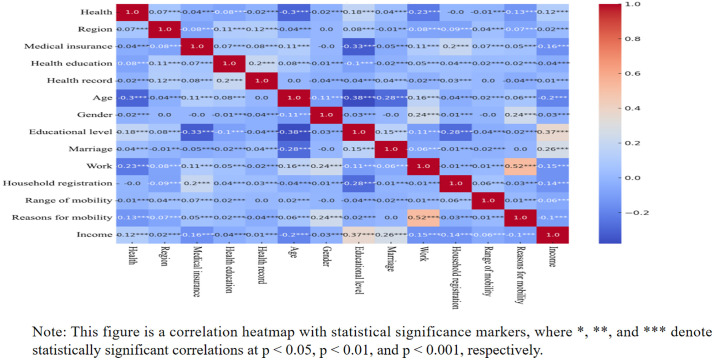
Correlation heatmap with significance.

The correlations among variables were visualized using a heatmap, with results shown in Fig 4. Pearson correlation analysis revealed that the linear correlation coefficients between migrant health and each variable were generally low (maximum |r| = 0.12), indicating limited explanatory power of individual independent variables for the target variable. Given the overall weak correlations between health and influencing factors, subsequent analyses may consider models capable of capturing complex relationships (e.g., random forests, gradient boosting). Additionally, correlations among independent variables were mostly low, though potential multicollinearity risks exist between work and migration reasons (r = 0.52) and education and income (r = 0.37), while linear dependencies among other variables were weak.

#### Multicollinearity test.

As indicated by the correlation analysis, several independent variables demonstrated moderate positive correlations, necessitating multicollinearity diagnostics prior to model construction. Accordingly, before conducting ordered logistic regression, this study first performed multicollinearity tests on different factors, with the results shown in Table 4. When tolerance > 0.1 or VIF < 10, it indicates that the correlation among the independent variables is relatively weak, and there is no multicollinearity. The independent variables pass the multicollinearity test. All variables in this study satisfied these criteria, confirming the absence of multicollinearity among the independent variables.

**Table 4:** Test for multicollinearity among independent variables.

Variables	VIF (<10)	Tolerance (>0.1)
Medical insurance	1.160	0.862
Gender	1.116	0.896
Age	1.000	1.000
Educational level	1.086	0.921
Marriage	1.003	0.997
Work	1.000	1.000
Household registration	1.001	0.999
Range of mobility	1.014	0.986
Reasons for mobility	1.011	0.989
Income	1.000	1.000
Health education	1.067	1.937
Health record	1.001	0.999
Region	1.214	0.806

#### Logistic regression analysis.

Although the correlation heatmap indicates generally weak linear correlations between independent and dependent variables, this does not preclude their significant effects within a logistic regression framework. Correlation coefficients only measure the strength of linear relationships, whereas logistic regression captures nonlinearities and interactions through the log-odds transformation. Therefore, the role of variables requires further evaluation through significance testing of regression coefficients. Prior to establishing machine learning models, ordered logistic regression was conducted, with migrant health as the dependent variable and multiple determinants as independent variables. Through multiple ordered logistic regression analysis, we examined the roles different factors play in influencing migrant health status, thereby conducting preliminary validation of the relationship between these determinants and health outcomes. The specific regression analysis results are shown in Table 5. Findings indicate that all selected characteristics significantly influenced the health status of migrants, with effects that were both positive and statistically significant. This indicates that there is a positive and significant relationship between different characteristics and the health of the migrant population.

**Table 5:** Ordered logistic regression analysis of health-related factors among migrant populations.

Variables		β	OR	Standard Error	Z-values	95%CI	P-values
Medical insurance	Not participating (reference)						
	Participation	0.182	1.199	0.027	8.02	1.148—1.255	<0.001
Gender	Female (reference)						
	Male	0.054	1.066	0.022	2.55	1.013—1.101	0.011
Age	15-30 (reference)						
	31-45	−0.706	0.494	0.014	−24.36	0.466 —0.522	<0.001
	46-60	−1.564	0.209	0.006	−49.66	0.197—0.023	<0.001
	61+	−2.351	0.095	0.004	−53.75	0.087—0.104	<0.001
Educational level	Illiteracy (reference)						
	Primary school	0.362	1.436	0.068	7.69	1.309—1.574	<0.001
	Junior high	0.682	1.978	0.092	14.73	1.807—2.166	<0.001
	High school	0.748	2.113	0.106	14.90	1.915—2.332	<0.001
	University	0.792	2.208	0.122	14.37	1.982—2.459	<0.001
Marriage	Unmarried (reference)						
	Married	0.130	1.139	0.037	3.99	1.068—1.214	<0.001
Work	Unemployment (reference)						
	Employment	0.529	1.699	0.049	18.06	1.604—1.799	<0.001
Household registration	Rural (reference)						
	Urban	0.162	1.175	0.064	2.97	1.056—1.308	0.003
Range of mobility	Cross county (reference)						
	Cross-city	0.083	1.086	0.025	3.67	1.039—1.136	<0.001
	Cross-province	−0.075	0.927	0.025	−2.83	0.880—0.977	0.005
Reasons for mobility	Work (reference)						
	Family	0.312	1.366	0.087	4.93	1.207—1.547	<0.001
	Other	0.083	1.087	0.035	2.59	1.021—1.157	0.009
Income	Lowest (<percentile 20) (reference)						
	Lower (percentile 20–39)	0.222	1.249	0.033	8.41	1.186—1.316	<0.001
	Middle (percentile 40–59)	0.301	1.351	0.037	10.89	1.280—1.427	<0.001
	Higher (percentile 60–79)	0.399	1.491	0.045	13.22	1.406—1.582	<0.001
	Highest (≥percentile 80)	0.418	1.519	0.051	12.47	1.422—1.622	<0.001
Health education	Not accepted (reference)						
	Accepted	0.496	1.643	0.084	9.69	1.486—1.816	<0.001
Health record	Not established (reference)						
	Established	0.128	1.137	0.057	2.57	1.031—1.254	0.010
Region	West (reference)						
	Central	0.106	1.112	0.027	4.48	1.061—1.166	<0.001
	East	0.327	1.386	0.033	13.58	1.322—1.453	<0.001
R^2^	0.122						
N	108669						

#### Comparative analysis of the importance of different factors affecting migrant health.

**Fig 5:**
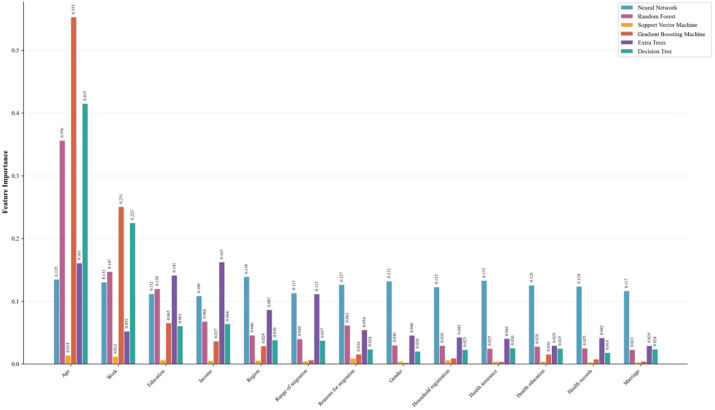
Features importance comparison across models.

Based on the logistic regression analysis, factors with significant effects were retained and further evaluated using machine learning techniques. Six algorithms were applied to compare feature importance, enabling the identification of key determinants of migrant health. The comparison is shown in Fig 5, which illustrates the distribution of feature importance across models. This bar chart illustrates the importance of various features in different models. It reveals that age (Age) is the most influential feature: age demonstrates significant importance in every model, particularly in the Gradient Boosting model, where its feature importance is highest (0.553), far exceeding that of other features. This indicates that age plays a central role in predicting the target variable. Furthermore, differences in feature importance across models reveal varying assessments of feature significance. For instance, in Support Vector Machine and Neural Network models, feature importance is distributed more evenly. In contrast, Gradient Boosting and Random Forest models assign higher weights to certain features like age and work, reflecting differences in feature selection approaches. In Random Forest and Extra Trees, features like occupation and income exhibit higher importance, indicating strong dependencies. Conversely, in Decision Tree models, characteristics such as marital status and health records appear more significant.

**Fig 6:**
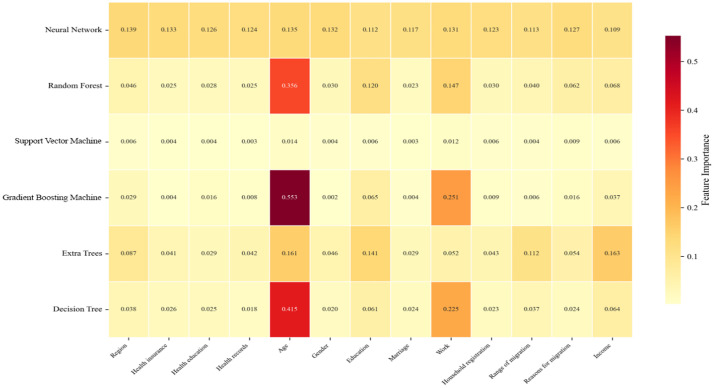
Feature importance heatmap across all models.

Fig 6 presents the Feature Importance Heatmap across all models, illustrating the importance scores assigned to each feature by different machine learning models. It is evident that age consistently receives high importance scores across most models, particularly in Gradient Boosting Machine and Random Forest models, where its importance scores significantly exceed those of other features at 0.553 and 0.356, respectively. This indicates that age significantly influences prediction outcomes across different models. Additionally, models exhibit substantial differences in feature sensitivity. For instance, the Support Vector Machine model shows minimal focus on all features, with importance scores approaching zero. This suggests the model may struggle to effectively identify relationships between features and the target variable. Random Forest and Gradient Boosting models place substantial emphasis on age, while neural networks and decision trees exhibit relatively uniform importance across features, revealing differing preferences in feature selection. Heatmaps reveal that certain features like Work and Income consistently rank highly across multiple models, indicating their stable contribution to model performance. In contrast, features such as Health Insurance and Health Records demonstrate lower importance, suggesting their limited influence within the models.

#### Performance evaluation of different machine learning models.

**Fig 7:**
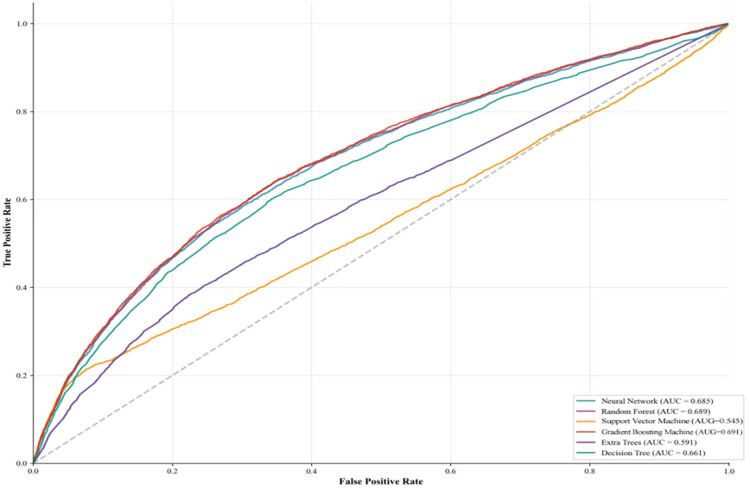
Comparison of ROC curves for different machine learning models.

**Fig 8:**
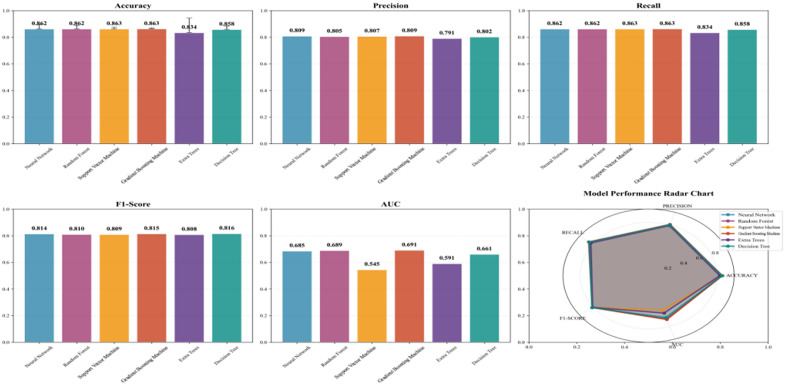
Performance comparison results across different models.

The predictive performance of six machine learning models was evaluated using the validation set. Among the six models, the Gradient Boosting Machine model achieved an AUC of 0.691, the Random Forest model achieved an AUC of 0.689, the neural network model achieved an AUC of 0.685, the Support Vector Machine achieved an AUC of 0.545, the Gradient Boosting model achieved an AUC of 0.691, the AUC for the hypertree model was 0.591, and the AUC for the decision tree model was 0.661 (Fig 7). Detailed results for other evaluation metrics across different models are shown in Fig 8.

**Fig 9:**
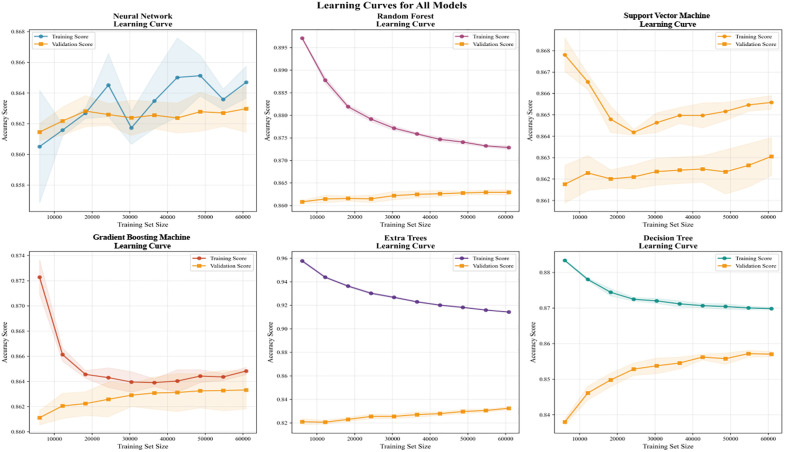
Learning curves for six machine learning models.

Fig 9 displays the learning curves of six machine learning models. These curves illustrate the relationship between training set size and training scores versus validation scores, aiding in analyzing the performance of different models during training. The results show that the Random Forest and Neural Network models exhibit relatively small gaps between training and validation scores, indicating strong adaptability and generalization. Hyper trees and decision trees exhibit significant gaps between training and validation scores, indicating they are prone to overfitting on training data and fail to generalize well on validation data. Support Vector Machine is sensitive to dataset size, performing poorly on small datasets but gradually improving on large ones. Gradient Boosting’s performance is similarly affected by dataset size, particularly exhibiting overfitting risks on small datasets, though its performance stabilizes as the training set grows. Based on the learning curve analysis across different models, Random Forests and Neural Networks demonstrate superior performance across most dataset sizes, particularly in terms of generalization capability and stability. These two models are recommended as the preferred choices for this task. Hypertrees and Decision Trees exhibit poor generalization on larger datasets and are prone to overfitting. Support Vector Machine and Gradient Boosting show weaker performance on small datasets but gradually reveal better learning efficiency and stability as the dataset size increases.

**Fig 10:**
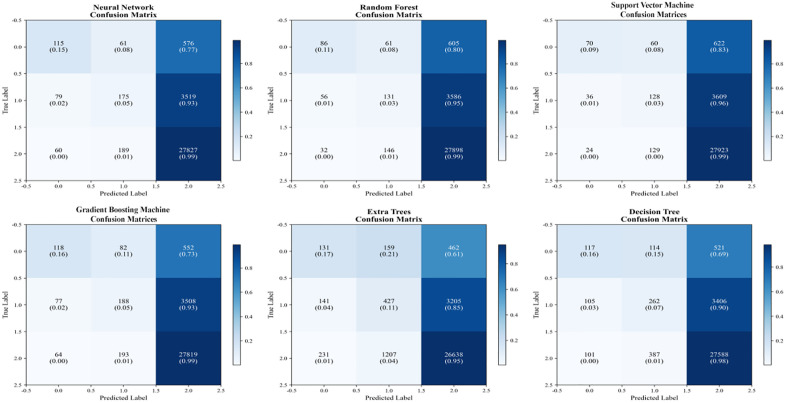
Comparison of confusion matrices across different machine learning models.

To further assess predictive performance, Fig 10 presents the confusion matrices for the six models, showing the relationship between predicted outcomes and actual labels across categories. The results reveal that Random Forest and Gradient Boosting exhibit relatively stable performance across all categories, particularly demonstrating high prediction accuracy and fewer misclassifications in Category 3. Neural Networks and Random Forest achieve exceptionally high prediction accuracy in Category 3, indicating strong generalization capabilities. Conversely, Support Vector Machine and HyperTree models show higher misclassification rates in certain categories, suggesting potential overfitting or suboptimal handling of specific categories. Decision trees and hypertrees exhibit elevated false positives and false negatives in certain categories, suggesting potential overfitting—especially when training datasets are small or imbalanced. Overall, random forests and gradient boosting demonstrate strong stability and low misclassification rates, performing excellently across all categories and proving suitable for practical applications.

**Fig 11:**
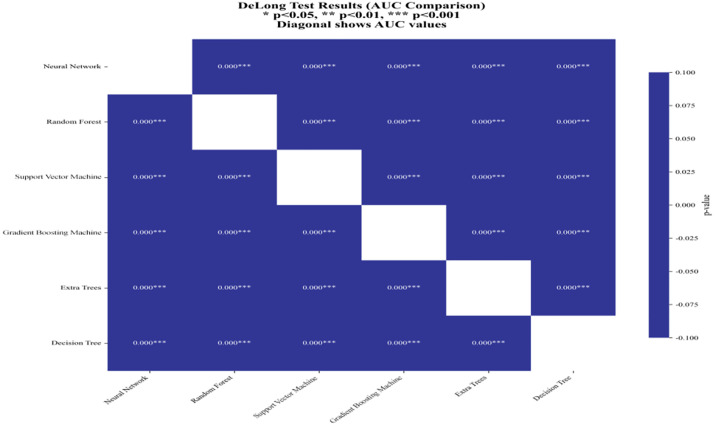
DeLong test results (AUC Comparison).

We also compared the AUC (area under the curve) values of different models using the DeLong test, with results shown in Fig 11. All pairwise comparisons yielded highly significant differences (p < 0.001), confirming meaningful distinctions in classification performance. Based on the AUC values and significance tests, Gradient Boosting and Random Forest demonstrated the best performance. They achieved higher AUC values and exhibited highly significant differences compared to other models. In contrast, the Decision Tree model had the lowest AUC value and showed very significant differences in comparison with other models, indicating its poor performance in this classification task.

**Fig 12:**
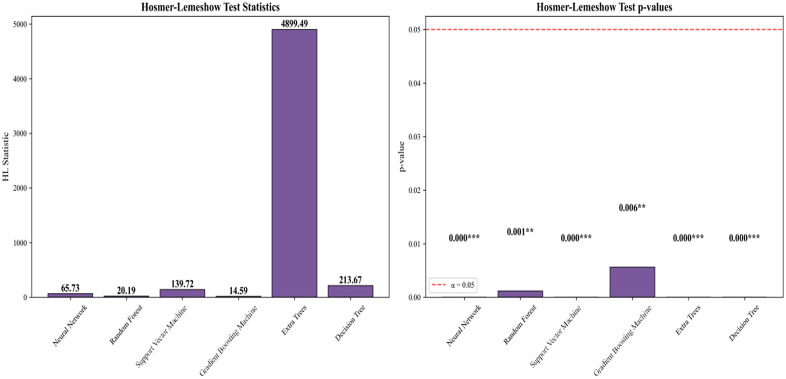
Hosmer-lemeshow test statistics and p-values.

Fig 12 further displays the statistical results of the Hosmer-Lemeshow test, which assesses model calibration by comparing predicted probabilities with observed outcomes. The Hosmer-Lemeshow test is commonly employed to assess a model’s accuracy in predicting probabilities; a higher test statistic and lower p-value indicate that the model fits the data well. Extra Trees demonstrates the strongest performance, exhibiting the highest Hosmer-Lemeshow statistic (4890.49) and the most significant p-value (0.000), indicating its superior fitting capability. Gradient Boosting also performs exceptionally well, featuring a high statistic (139.72) and a significant p-value (0.000), suggesting robust fitting effectiveness. Neural Network and Random Forest also demonstrate good goodness-of-fit, with their statistics and p-values indicating strong performance on this task. Support Vector Machine and Decision Tree exhibit poorer fitting capabilities, particularly Support Vector Machine, which has the lowest statistic and a higher p-value, suggesting certain fitting issues with this model.

Based on the comprehensive evaluation, although the AUC value of Gradient Boosting (0.691) is slightly higher than that of Random Forest (0.689), this difference is relatively small. In practice, model selection should not rely solely on AUC; additional factors such as stability, computational efficiency, overfitting risk, and interpretability must also be considered. Random Forest demonstrated excellent performance in AUC, confusion matrices, and the Hosmer-Lemeshow test, exhibiting high accuracy and low misclassification rates. Furthermore, it showed strong adaptability to both training and validation sets, indicating good generalization capabilities. Even with Gradient Boosting’s marginally higher AUC, Random Forest delivered more balanced performance, particularly regarding overfitting and model training stability. The neural network model showed good performance in the confusion matrix but may encounter overfitting issues in certain scenarios, while exhibiting some fluctuations in the learning curve. The Extra Trees model exhibits strong fitting capability. However, it underperforms relative to Random Forest and Gradient Boosting in other evaluation metrics (e.g., AUC, confusion matrix). Support Vector Machine and Decision Tree demonstrate relatively average performance, showing significant gaps, particularly in AUC and goodness-of-fit. Therefore, considering overall performance and stability, Random Forest remains the recommended optimal model.

**Fig 13:**
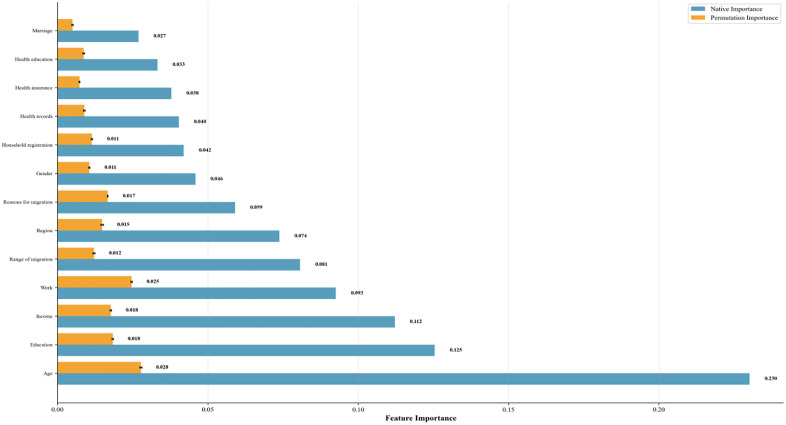
Comparison of native and permutation feature importance in random forest model.

Fig 13 displays the Native Importance and Permutation Importance results calculated from the top-performing Random Forest model. Age consistently ranks as the most important variable. Age holds the highest importance value in Native Importance (0.230) and maintains its top position in Permutation Importance (0.083), highlighting its central role in predicting migrant health outcomes. Education and Income rank highly in native importance (0.125 and 0.112, respectively), but their values decrease significantly in permutation importance (both around 0.018), suggesting they are frequently utilized in random forest splits. Employment status (Work) maintained moderate importance in both methods (Native = 0.093, Permutation = 0.025), indicating a stable influence of employment factors on health. Variables related to migration (including migration scope, migration reasons, and region) showed moderate importance in the native analysis (0.07–0.09) and retained some contribution in the permutation analysis (0.017–0.022). In contrast, variables such as marital status, gender, household registration, health education, medical insurance, and health records showed relatively low importance, with contribution values approaching zero in the permutation analysis. This indicates the limited predictive utility of these factors for health status. Therefore, the feature importance analysis highlights age, education, income, and employment as the dominant drivers of migrant health, with age being the most critical determinant. Migration-related variables play secondary roles, while other sociodemographic characteristics exert only minor effects.

#### Explainable model analysis using SHAP.

**Fig 14:**
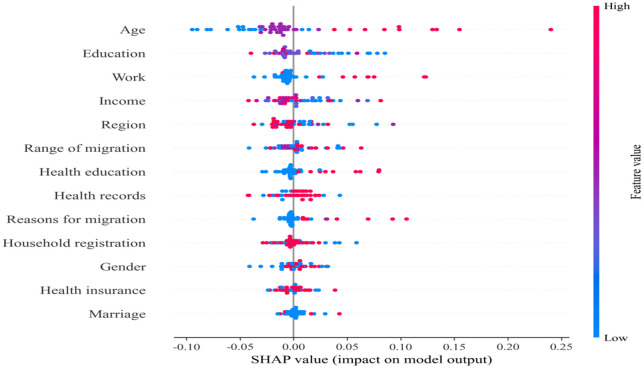
The SHAP plot illustrates the relative importance of different factors in influencing the health status of the floating population.

**Fig 15:**
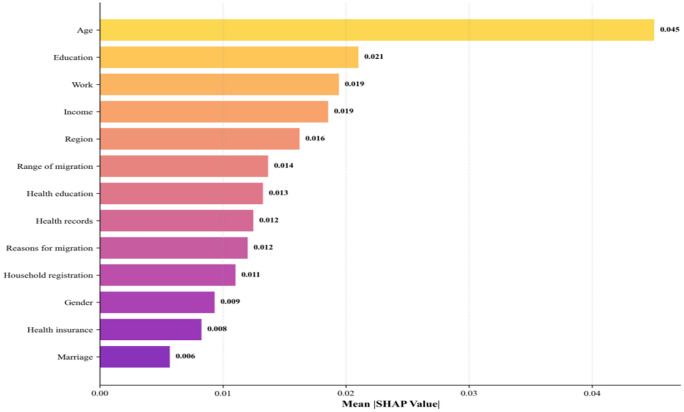
SHAP feature importance (Mean).

To further enhance interpretability, we employed SHAP analysis. SHAP values provide a unified metric for feature importance, enabling us to understand each feature’s contribution to the model’s prediction outcomes. The SHAP analysis was conducted on the best-performing random forest model, which demonstrated superior performance in our preliminary evaluations. We computed SHAP values across the entire dataset to identify the global importance of features, illustrating the extent to which each feature influences the “health” target variable. The SHAP plot reveals the magnitude and direction of each feature’s contribution to predicting health levels, with each point representing a sample. The color gradient from red to blue indicates feature value magnitude, where red denotes high values and blue denotes low values. Fig 14 summarizes the SHAP analysis, illustrating how each feature influences migrant health levels. Fig 15 displays feature importance through the mean absolute SHAP value. Based on these findings, health levels typically decline with increasing age, with higher age values pushing health toward poorer outcomes—indicating older individuals are often predicted as unhealthy or barely healthy. Educational attainment ranks as the second most influential factor. Higher education typically correlates with improved health awareness and lifestyle choices, leading to better health outcomes. Additionally, income and employment status play significant roles in health levels. Individuals with higher incomes generally enjoy better health conditions, while the impact of employment status on health is more complex. Its SHAP values exhibit a wide distribution, indicating that different employment statuses have varying effects on health levels, though employment itself is often associated with better health. The impact of region on health status is complex, with SHAP values distributed relatively uniformly and no clear unified trend, indicating that regional differences do not significantly influence health status. Mobility range has a minor effect on health status, with SHAP values distributed close to zero, suggesting its role is relatively weak. Health education has some influence on health status, though relatively small. Health records exert a certain impact, with good health records (red dots) typically associated with better health conditions. However, the relatively narrow distribution of SHAP values for this feature indicates its relatively minor contribution to health status. Other features, such as marital status, medical insurance, gender, household registration, and reasons for mobility, exert the weakest influence in the model. Therefore, age, education level, income, and employment are key determinants of migrant health, while other characteristics play more limited and supplementary roles.

#### Feature sensitivity analysis.

**Fig 16:**
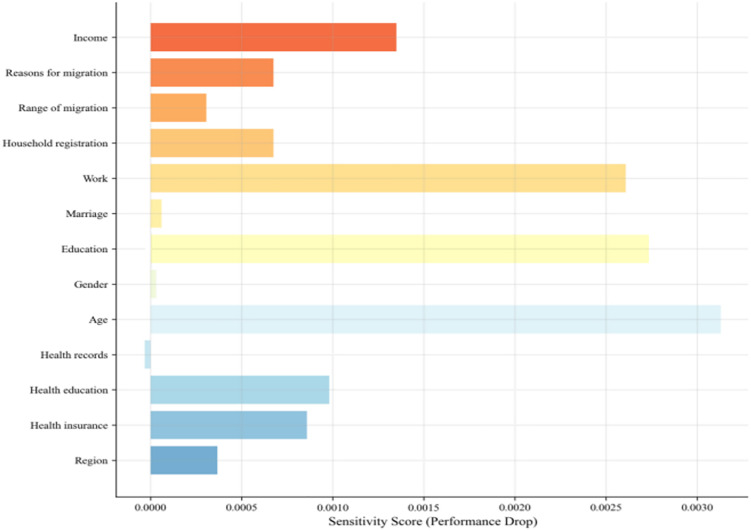
Feature sensitivity analysis.

To evaluate the impact of individual features on model predictive performance, this study conducted a feature sensitivity analysis. Fig 16 presents the feature sensitivity analysis diagram, which assesses the influence of each feature on model performance. The sensitivity score in the diagram indicates the degree of model performance decline after removing a specific feature, reflecting its importance for model prediction. The feature sensitivity analysis revealed that age, education, occupation, and income are the most influential features for the health status prediction model. Removing these features significantly reduces model performance, indicating their critical importance. In contrast, health records, migration reasons, household registration, health education, and health insurance have a relatively minor impact on model performance. Features like gender, marital status, region, and education level contribute even less to the model’s accuracy.

## Discussion

### Analysis of the health status of China’s migrant population

This study aims to analyze the health levels of China’s migrant population and their key factors. The findings contribute not only to theoretical research on migrant health but also provide practical support for improving health policies for this population, thereby promoting their health outcomes. Findings reveal that China’s migrant population generally enjoys high health levels, with 97.7% reporting good health status. This aligns with their age distribution, where 96.2% fall within the working-age population (15–60 years old). These finding aligns with previous studies. For instance, research utilizing 2015 and 2017 China General Social Survey (CGSS) data examined the impact of internet usage on the physical and mental health of China’s migrant population. Results indicated mean physical and mental health scores of 3.923 and 3.858, respectively, clearly reflecting the healthy state of this demographic [58]. Another study utilizing 2017 China Migrant Population Dynamic Monitoring Survey data analyzed the health status and equity among migrant workers. Results indicated that 97.29% of migrant workers self-reported being in a healthy state, while 2.71% reported being unhealthy, confirming their generally favorable health condition [59]. Despite differences in databases, both studies confirm that China’s migrant population enjoys relatively high health levels. This also underscores the need for continuous improvement in the health status of the migrant population, as health serves as the foundation for better leveraging their human capital advantages [60].

When compared with international migrant populations, China’s migrant population shares common characteristics faced by migrant groups while also exhibiting unique features. In terms of common characteristics, the overall health levels of China’s migrant population, like those in other countries, are generally lower than those of the local population. This special group typically engages in labor-intensive occupations, faces poor living conditions, has weak social support systems, and encounters barriers to accessing healthcare services. The uniqueness of China’s migrant population in terms of health status manifests in two aspects. First, China’s urban-rural dual household registration system makes it difficult for the migrant population to obtain the same education, medical insurance, housing, and public health services as residents in cities. This is relatively uncommon in other countries and represents a key institutional root cause of health issues among China’s migrant population. Second, unlike many countries where migration is primarily transnational, China’s migrant population consists mainly of internal migrants. These shared characteristics indicate that while public health policies implemented for migrant populations in other countries can provide valuable reference points for further safeguarding the health of China’s migrant population, the unique circumstances of China’s migrant population also dictate that the optimization of health protection policies must be grounded in China’s specific realities.

### Key factors influencing the health of migrant populations

This study employs Anderson’s Health Service Utilization Model to identify factors affecting migrant health across three dimensions: propensity characteristics, enabling resources, and contextual features. Machine learning methods were applied to comprehensively evaluate these factors. Consistent with prior research [61], the findings indicate that migrant health is influenced by multiple factors. However, unlike prior studies, this study not only identifies the relationship between these factors and migrant health but also employs multiple machine learning models to measure the relative importance of each factor. This approach helps pinpoint the key determinants of health among China’s migrant population, thereby providing more scientific evidence for identifying health risks and optimizing health security policies for this group.

The findings reveal that age, education, employment, and income are the key determinants of migrant health. Among these, age is the most critical factor affecting migrant health status. Its impact manifests as a decreasing probability of maintaining better health levels with advancing age. This aligns with existing research findings from China and other countries, which indicate that as people age, physiological functions gradually decline, metabolism slows, and immune system efficiency decreases, leading to increased susceptibility to disease [62–65]. Migrant populations typically lack stable long-term residences and work environments. They also face disadvantages in income, education, social security, and public health services. The instability and vulnerability stemming from this mobility exacerbate the health impacts of physiological decline. Furthermore, when facing health issues, migrant populations often cannot access the same level of health protection as residents, making it difficult to receive timely and effective prevention and treatment. Diseases may worsen due to the neglect of early symptoms. At the same time, migrant populations of different age groups may also have differences in health needs and risk exposure. When improving health policies for migrant populations in the future, it is also necessary to develop stratified intervention strategies based on differences in age structure.

Education was also identified as a key determinant, with higher educational attainment correlating with improved health outcomes. This finding is consistent with previous research. For example, a Chinese study of 1,283,774 individuals born between the 1940s and 1970s demonstrated significantly higher all-cause mortality risks among those with lower education levels [66]. A U.S. study examining the relationship between education and mortality found that participants with higher education levels exhibited lower mortality risks, and those attaining higher education tended to age more slowly and live longer [67]. Norwegian researchers concluded that education extends lifespan: each additional year of education reduces all-cause mortality risk by 1.9%, demonstrating a robust correlation independent of age, gender, location, and socio-demographic background [68]. Educational attainment objectively reflects an individual’s capacity to access health information, acquire health knowledge, and master health-related skills. Higher education not only enhances migrant populations’ awareness of their own health but also provides them with broader access to health-related knowledge and stronger health consciousness, thereby facilitating better health outcomes. However, this study reveals that the migrant population generally has low educational attainment. This may hinder their ability to accurately assess their own health status, preventing them from accessing timely health services. Yet, as a key propensity factor in Anderson’s Health Utilization Model, while education significantly impacts migrant health, it is inherently difficult to change in the short term. Its influence is thus more evident as a background variable. The limited scope for policy intervention at the educational attainment level dictates that future optimization of health security policies for the migrant population should focus more on enhancing health levels by improving enabling resources and meeting demand factors. This could include tailored health education programs targeting common diseases among migrants (such as hypertension and occupational illnesses). Consequently, even within the specific group of migrants with lower educational attainment, their health security capabilities can be enhanced by reducing barriers to accessing enabling resources and improving demand factors.

Employment exerts a substantial influence on migrant health. Having stable employment is strongly associated with better outcomes, consistent with previous studies [60, 61]. This may be because employment typically provides migrants with a stable income source, enabling them to access more health resources promptly to safeguard their well-being and avoid neglecting health issues due to excessive financial pressure. Additionally, employed migrant workers typically gain access to basic medical coverage through workplace or social insurance programs [69], enabling them to receive timely treatment when ill and reducing the risk of health issues worsening. Unemployed migrants, however, often lack such medical coverage, preventing them from accessing equivalent healthcare resources and reimbursement benefits, thereby increasing their health risks. Furthermore, employment not only provides economic support but also fosters a sense of social belonging and self-worth among migrant workers [70]. Employment fosters social recognition and self-actualization, contributing to improved mental health and reduced incidence of psychological issues like anxiety and depression. Unemployed or underemployed migrant workers, however, often experience social isolation, loneliness, and helplessness, which adversely affect their mental well-being.

Income similarly affects health outcomes, with higher income linked to better health through increased capacity for disease prevention, treatment, and adoption of healthy behaviors. They can access health information through more channels [71,72] and adopt scientific preventive measures (such as regular check-ups and balanced diets) to maintain or improve their health status. In contrast, low-income groups may neglect health management behaviors due to a lack of time, funds, or awareness, leading to delayed detection of health issues. Furthermore, low-income migrant workers may lack sufficient health education and health management knowledge, preventing them from taking timely and effective measures when health problems arise. The analysis results of this study also indicate that the income of migrant workers is mostly below the middle level. Therefore, in health security, priority attention should be given to low-income migrant workers.

### Analysis of the application value of machine learning methods in migrant population health management

This study combines machine learning algorithms with large-scale cross-sectional survey data specifically targeting China’s migrant population to construct an analytical model of health determinants among this group. Compared to traditional linear regression-based models, machine learning algorithms effectively mitigate distortions caused by noise interference in real-world data. Through nonlinear and highly interactive variable combinations, these algorithms enable the construction of more complex and precise predictive models. By applying multiple algorithms, the study confirmed the robustness and accuracy of machine learning in identifying key predictors of migrant health. All algorithms demonstrated high accuracy, confirming the effectiveness of machine learning in identifying critical predictors for this vulnerable population.

The application of SHAP values in this study enhances model interpretability, clearly revealing how each feature influences predictions. This aids policymakers in better understanding protective and risk factors affecting migrant health and developing more effective intervention strategies. SHAP analysis confirmed the significant impact of characteristics such as age, occupation, education level, and income on migrant health outcomes. These findings deepen existing literature on migrant health and highlight the necessity for targeted preventive measures in these areas. Integrating SHAP analysis into research provides a robust framework for understanding the factors promoting and hindering migrant health. By offering global interpretations, SHAP values enhance the transparency and credibility of machine learning models, ultimately supporting better decision-making in migrant health protection.

Currently, machine learning methods are widely used in medicine, public health, and other fields for predicting various disease risks, demonstrating significant value [73,74]. However, its application to migrant populations remains limited. Existing studies have primarily focused on predicting reproductive behaviors using artificial neural networks and Naïve Bayes models [75], or forecasting job retention and satisfaction among inter-state migrants with decision trees, random forests, and gradient boosting algorithms [76].

To our knowledge, this study represents the first application of machine learning models to representative survey data on China’s migrant population, aiming to identify and rank factors influencing migrant health. Migrant populations represent a distinct group both in China and globally. Their vulnerable positions in employment, education, and income expose them to heightened health risks. Therefore, identifying key determinants of migrant health and tailoring health protection policies accordingly holds significant importance for reducing health risks and improving health outcomes among this population. Machine learning methods not only assist in identifying determinants but also provide new opportunities for health management. This includes enabling early intervention through precise disease risk prediction, providing personalized health management plans based on individual characteristics, and facilitating dynamic health monitoring and rapid response through real-time tracking technologies. Collectively, these approaches can effectively elevate the overall health status of the migrant population.

### Policy recommendations

Based on the findings, the following recommendations are proposed. First, develop tiered intervention strategies tailored to age-based differences. Migrant populations across different age groups not only engage in distinct occupations but also possess varying capacities to withstand health risks. Therefore, tiered intervention strategies should be formulated according to age-based differences. For instance, big data platforms and health information systems can be used to analyze age-stratified health risks. This facilitates the exploration of age-stratified public health services: prioritizing occupational health for young migrants, chronic disease screening for middle-aged individuals, and medical accessibility with long-term care for the elderly. Such an approach enables more precise and actionable health policies. Second, optimize the working environment and employment security. Migrant workers often endure poor labor conditions and elevated health risks. These risks can be mitigated through stricter occupational health and safety regulations, expanded workplace health examinations, and increased awareness of occupational disease prevention. Employers should be encouraged to legally sign labor contracts with migrant workers, explicitly outlining their employment rights to strengthen job stability and reduce anxiety and uncertainty. Third, enhance the health literacy. Health departments can regularly organize experts in medicine, public health, and related fields to visit migrant workplaces, providing health education and medical check-ups. Public health policies should be promoted through short videos, social media platforms, and community health stations to improve understanding of health security policies among migrants with lower education levels. Fourth, strengthen public health emergency management and social support systems for migrant populations. Their socioeconomically vulnerable position means public health crises pose greater health challenges, often leaving them resource-deprived during emergencies—particularly lacking timely warnings and responses during outbreak initial stages. Inadequate social support systems further hinder access to essential assistance during crises. Establish robust health monitoring and early warning systems for the migrant population to promptly assess their health status and needs. Public health emergency management must fully consider the unique characteristics of migrants, developing targeted contingency plans and measures. This includes establishing comprehensive health monitoring and early warning systems to track their health conditions and requirements; providing essential psychological, material, and social support in areas with high migrant concentrations.

## Conclusion

This study identified several key factors influencing the health of China’s migrant population, including employment, educational attainment, income, and age. Given their significant impact, future efforts to optimize health protection for migrants should prioritize these factors. Targeted interventions in these areas are likely to be more effective in reducing health disparities and addressing barriers that place this group at greater risk of poorer health outcomes.

### Limitations

Several limitations should be acknowledged. First, the data were derived from secondary sources within a publicly available dataset (the latest version of which was released in 2018), implying a degree of data lag. Future analyses should utilize more recent data sets, such as the China Labor-force Dynamic Survey, could further explore emerging patterns in migrant health. Additionally, the data employed in this study were from a large cross-sectional survey, which was used to examine the relationship between migrant health and key determinants in China. However, causal inferences cannot be drawn from existing cross-sectional data. Future research with richer datasets—such as the China Labor-force Dynamic Survey or longitudinal studies—could further explore the temporal evolution of key health determinants among migrant populations and the dynamic shifts in health equity. Third, while the CMDS possesses strong representativeness, it exists limitations, such as respondent subjectivity in questionnaire responses and uneven regional coverage. Fourth, given the complexity of health determinants, this study may have omitted other factors influencing migrant health (e.g., disease type, severity, and healthcare utilization). Future research should consider incorporating these potential factors into analyses. Finally, while the study discusses the relationship between public health crises and migrant health based on existing research, data limitations prevented an empirical analysis of this relationship. As relevant data become available, future studies should conduct quantitative analyses to validate and extend these findings.
